# Dual-frequency intraductal ultrasonography: a breakthrough in biliopancreatic imaging during endoscopic retrograde cholangiopancreatography

**DOI:** 10.1055/a-2578-2854

**Published:** 2025-05-06

**Authors:** Yao Lu, Xiaoyan Lv, Shun He

**Affiliations:** 1 71041 Department of Endoscopy, Cancer Hospital Chinese Academy of Medical Sciences, Beijing, China

Intraductal ultrasonography (IDUS) is a reliable procedure for evaluating the biliopancreatic duct during endoscopic retrograde cholangiopancreatography (ERCP) 1 2 ; however, conventional high frequency IDUS is limited by its penetration depth 3 . This case highlights a novel dual-frequency IDUS probe that overcomes this limitation ( Fig. 1 ; Video 1 ).

**Fig. 1:**
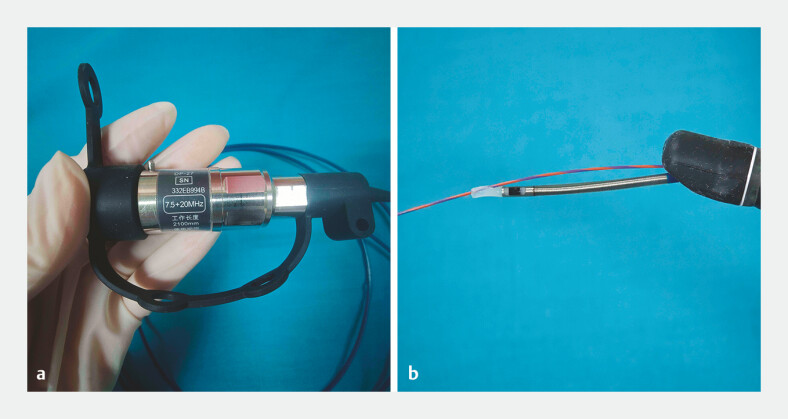
Photograph of the dual-frequency intraductal ultrasonography probe showing: **a** the probe, which features two frequencies (20 MHz and 7.5 MHz) that are switchable via the main engine; **b** the probe tip, which has an outer diameter of 2.5 mm.

A novel dual-frequency intraductal ultrasonography probe is used to evaluate biliopancreatic disease during endoscopic retrograde cholangiopancreatography.Video 1

A 60-year-old woman was referred to our hospital with jaundice. Magnetic resonance imaging (MRI) revealed a pancreatic head mass with distal bile duct obstruction ( Fig. 2 ). Laboratory tests showed she had a serum total bilirubin of 330 μmol/L and a CA19-9 of 146 U/mL, and a preliminary clinical diagnosis of pancreatic head cancer was made.

**Fig. 2:**
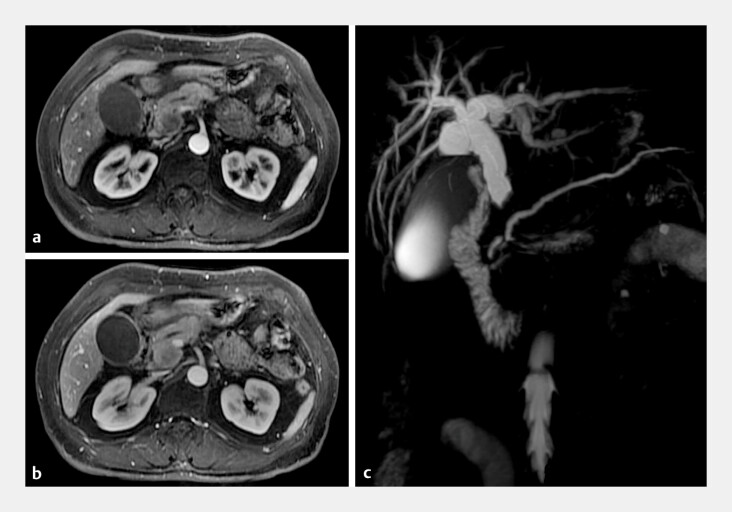
Enhanced magnetic resonance imaging and magnetic resonance cholangiopancreatography (MRCP) images showing: **a** in arterial phase, mild enhancement of the pancreatic head lesion; **b** in portal phase, progressive enhancement; **c** on MRCP, a distal bile duct stricture with upstream bile duct dilatation and slight pancreatic duct dilatation.

Endoscopic retrograde cholangiography (ERC) revealed a defect in the distal bile duct on contrast injection. To determine the nature of this biliary stricture, a novel IDUS probe with dual frequencies of 20 MHz and 7.5 MHz (DP-27, 7.5+20 MHz; Innermed, Shenzhen, China) was advanced over guidewires into the pancreatic duct and bile duct, which were scanned using the pull-back method. Using the 20-MHz frequency, the IDUS scan showed the pancreatic duct and proximal surrounding structures ( Fig. 3 **a** ). On switching to 7.5 MHz, the far-field resolution significantly improved, allowing visualization of the complete pancreatic contour and parenchyma ( Fig. 3 **b** ). The pancreatic head appeared as a heterogeneous hypoechoic region without any evident tumorous lesions. A subsequent 20-MHz scan of the intrapancreatic bile duct revealed a circular symmetrical wall thickening, with a smooth outer margin ( Fig. 3 **c** ). A switch to 7.5 MHz confirmed no evidence of an extrinsic lesion causing compression ( Fig. 3 **d** ).

**Fig. 3:**
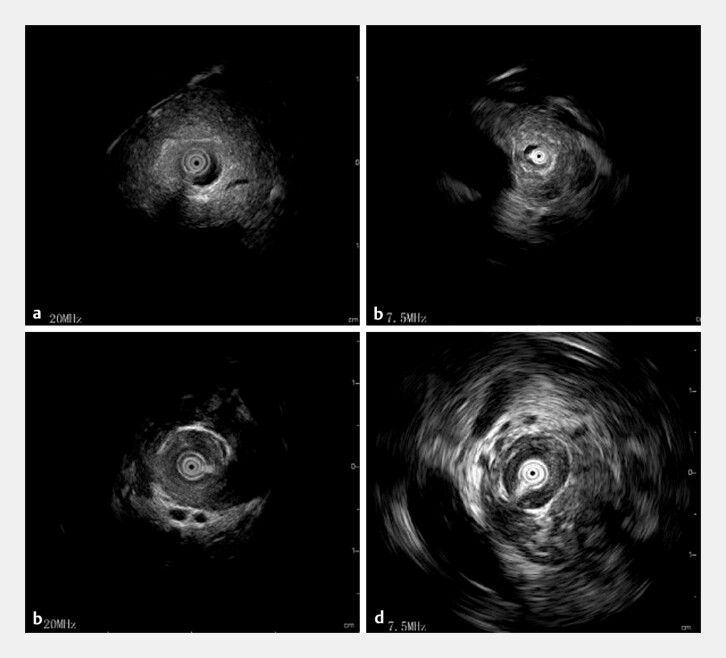
Images during dual-frequency intraductal ultrasonography (IDUS) showing; **a** the appearance on 20-MHz IDUS of the pancreatic duct; **b** on 7.5-MHz IDUS, a high resolution image of the pancreatic parenchyma; **c** on 20-MHz IDUS, intrapancreatic biliary wall thickening; **d** on 7.5-MHz IDUS, detailed periductal structural information.

The IDUS images were therefore used to make a diagnosis of autoimmune pancreatitis and IgG4-related sclerosing cholangitis (IgG4-SC), which was confirmed by finding an elevated serum IgG4 level and on endoscopic ultrasound-guided fine-needle aspiration (EUS-FNA). Following glucocorticoid therapy, the patient’s symptoms resolved, and imaging showed significant improvement. This dual-frequency IDUS technology offers enhanced diagnostic capability and can be seamlessly integrated into routine ERCP procedures, significantly reducing diagnostic delays and improving patient management.

Endoscopy_UCTN_Code_TTT_1AS_2AD

## Notes

Endoscopy E-Videos https://eref.thieme.de/e-videos
*E-Videos* is an open access online section of the journal *Endoscopy* , reporting on interesting cases and new techniques in gastroenterological endoscopy. All papers include a high-quality video and are published with a Creative Commons CC-BY license. Endoscopy E-Videos qualify for HINARI discounts and waivers and eligibility is automatically checked during the submission process. We grant 100% waivers to articles whose corresponding authors are based in Group A countries and 50% waivers to those who are based in Group B countries as classified by Research4Life (see: https://www.research4life.org/access/eligibility/ ). This section has its own submission website at https://mc.manuscriptcentral.com/e-videos .
